# Innovation: ice cream in the recovery room rules out chylothorax after thoracic lymphadenectomy and affords same-day chest tube removal

**DOI:** 10.3389/fsurg.2024.1457561

**Published:** 2024-08-13

**Authors:** Robert J. Cerfolio, Ashley J. McCormack

**Affiliations:** Department of Cardiothoracic Surgery, NYU Langone Health, New York, NY, United States

**Keywords:** chylothorax, thoracic lymphadenectomy, ice cream, chest tubes, pulmonary resection

## Abstract

**Objectives:**

Early removal of chest tubes reduces pain and morbidity. This study aimed to remove chest tubes immediately after robotic pulmonary resection with complete thoracic lymphadenectomy by administering ice cream to rule out chylothorax.

**Methods:**

This quality improvement study utilized prospectively gathered data from one thoracic surgeon. Patients were given 3.6 fl oz of ice cream in the recovery room within 1 h after their operation. Chest tubes were removed within 4 h if there was no chylous drainage and air leak on the digital drainage system.

**Results:**

From January 2022 to August 2023, 343 patients underwent robotic pulmonary resection with complete thoracic lymphadenectomy. The median time to ingest the ice cream was 1.5 h after skin closure. The incidence of chylothorax was 0.87% (3/343). Two patients were diagnosed with chylothorax after consuming ice cream within 4 h of surgery. One patient, whose chest tube remained in place due to an air leak, had a chylothorax diagnosed on postoperative day 1 (POD1). All three patients were discharged home on POD1 with their chest tubes in place, adhering to a no-fat, medium-chain triglyceride diet. All chylothoraces resolved within 6 days. None of the remaining patients developed chylothorax postoperatively with a minimum follow-up period of 90 days.

**Conclusions:**

Providing ice cream to patients after pulmonary resection and complete thoracic lymphadenectomy is an effective and reliable technique to rule out chylothorax early in the postoperative period and facilitates early chest tube removal. Further studies are needed to ensure that this simple, inexpensive test is reproducible.

## Introduction

Chylothorax is a known and potentially serious complication after pulmonary resection with complete thoracic lymphadenectomy. Its incidence after pulmonary resection is approximately 1%–2.5% (1–4). Lymphatic disruption after robotic thoracic lymphadenectomy for lung cancer, unlike esophagectomy for esophageal cancer, is not caused by injury of the main thoracic duct but rather by disruption of collaterals, most commonly at the base of mediastinal N2 lymph nodes, specifically stations 2R, 4R, and 7 (1, 5). In our experience, we have noted the 2R lymph node bed to be the most common location followed by the subcarinal area. Chylothorax is often suspected with an unexplained high-volume serous chest tube output and/or a white, milky chest tube drainage. The diagnosis is confirmed by an elevated triglyceride level of greater than 110 mg/ml in the chest tube effluent (1, 3). A fatty meal or diet is given as a provocative test to detect a chyle leak.

In this study, we aimed to rule out chylothorax immediately after robotic pulmonary resection with complete thoracic lymphadenectomy by administering ice cream in the recovery room to hasten chest tube removal. Our rationale was that by ruling out chylothorax sooner, the chest tubes could be removed in patients without air leaks, leading to an improved patient experience with reduced pain and morbidity.

## Materials and methods

### Study design

This was a quality improvement study that leveraged prospectively gathered data from a single institution and a single thoracic surgeon (RC). All patients who underwent robotic pulmonary resection, either lobectomy, segmentectomy, or wedge resection, by the surgeon in this series were included in this study. The study design, including a waiver of patient consent, was approved by the Institutional Review Board at NYU Langone Health #i23-01085. The primary outcome was the successful ruling out of chylothorax within 4 h after the patient consumed ice cream in the recovery room. The secondary outcomes included chylothorax diagnosis before discharge and/or within 90 days of surgery, thoracentesis or reinsertion of a chest tube within 90 days of surgery, and minor and major morbidity attributed to a chylothorax.

### Perioperative and postoperative management

All patients were evaluated, as we have previously described, by computed tomography scan, integrated positron emission tomography, pulmonary function testing, and stress test in selected patients (6). Lung resection was conducted with the da Vinci Xi surgical system (Intuitive Surgical, Sunnyvale, CA, USA) through a four-arm port placement and an assist port as we have previously reported using bipolar energy instruments for dissection (7). We performed a complete thoracic lymphadenectomy for all lung resections, regardless of pathology. This includes resection of lymph node stations 10, 11, 12, 13, 9, 8, and 7 bilaterally, with the addition of stations 2R and 4R for right-sided resections and stations 5 and 6 for left-sided resections. After the operation, a single 24-French chest tube was inserted in the access port and placed apically and posteriorly then connected to a digital drainage system, Thoraguard (Centese, Omaha, NE, USA).

Upon arrival in the recovery room, a portable chest x-ray (CXR) was obtained. The patients were given 106 ml (3.6 fl oz) of Häagen-Dazs ice cream with approximately 15 g of total fat as a provocative test for chylothorax in the recovery room as soon as they were able to tolerate food by mouth. If the patient was nauseous, ice cream was given as soon as they were able to ingest it. Appropriate modifications were made for patients with dietary restrictions such as lactose intolerance or diabetes mellitus by providing lactase enzyme or low-sugar ice cream. Chest tube removal was scheduled after the patients consumed the ice cream and after waiting a minimum of 1 h. The chest tube was removed 2–6 h postoperatively if the following criteria were met: (1) chest tube effluent was not cloudy or milky, (2) patients were hemodynamically stable with oxygen saturations stable at their baseline, (3) CXR showed either complete lung expansion or a pleural space deficit not larger than predicted or expected (8), and (4) there was no air leak on the digital drainage system, which was defined by a leak of less than 50 ml/min and only negative numbers on the pleural assessment test. Chest tubes were removed irrespective of the volume drained, as we have previously reported (9).

If the effluent was suspicious for chyle, the chest tube was kept in place, and the pleural fluid was sent for triglyceride level analysis. A diagnosis of chylothorax was confirmed if the triglyceride level was greater than >110 mg/ml (3). If the triglyceride level was not elevated, the patient was encouraged to consume more ice cream, and the chest tube effluent was retested. Per our previously published algorithm (1), all chylothoraces were treated medically with a modified diet consisting of no fat and medium-chain triglycerides. Patients diagnosed with chylothorax were discharged home by 8 a.m. on postoperative day (POD) 1 with a chest tube in place attached to a digital drainage system (10, 11). They received cephalexin 250 mg orally once daily as a subclinical prophylactic antibiotic dose until the tube was removed. Chest tubes were removed on POD14 irrespective of an air leak, as we have previously reported (12).

### Care at home

The patients and their caregivers were taught how to use the digital air leak device and were instructed to send a text to the surgeon with daily updates about the quality and quantity of drainage and send pictures and/or videos of the drainage system. In addition, they were instructed to purchase a pulse oximeter to record their heart rate and oxygen saturation levels at home and then to send a text with this information to the surgeon. The patients presented to the surgeon's office for chest tube removal once the chylous drainage resolved or the quantity minimized to less than 200 ml per day after having a fatty meal at home as a challenge test the day before returning to the office.

Major and minor adverse events and readmissions within 30 days of the operation were included in the perioperative data. Minor and major complications were defined as Grade I–II and Grade III or higher, respectively, based on the Clavien–Dindo classification system.

### Statistical analysis

Descriptive analyses were used to report the patients’ baseline characteristics, intraoperative course, and postoperative outcomes. Categorical variables were reported as frequencies and percentages. Continuous non-normally distributed variables were reported as median with interquartile range. In addition, sensitivity, specificity, predictive values, and accuracy were calculated to evaluate the diagnostic performance of the ice cream challenge test. Statistical analyses were performed using IBM SPSS Statistics, version 26.0 (IBM, Armonk, NY, USA).

## Results

From January 2022 to August 2023, 343 patients underwent a robotic pulmonary resection with complete thoracic lymphadenectomy by one surgeon (Table 1). Of these patients, 178 underwent lobectomy (51.9%), 106 underwent segmentectomy (30.9%), and 59 underwent wedge resection (17.2%). The study cohort was comprised of 209 women (60.9%) with a median age of 68 years (Table 2).

**Table 1:** Patient characteristics.

Variable	All patients *N* = 343
Age, years, median (IQR)	69 (62–75)
Sex, *n* (%)	
Female	209 (60.9)
Male	134 (39.1)
BMI, kg/m^2^, median (IQR)	25 (22–28)
Neoadjuvant treatment, *n* (%)	27 (7.9)
FEV_1_%, median (IQR)	94 (82–105)
D_LCO_ %, median (IQR)	79 (68–91)
ECOG score, *n* (%)	
0	222 (64.6)
1	115 (33.5)
2	2 (0.58)
3	4 (1.2)
Hypertension, *n* (%)	183 (53.4)
Hyperlipidemia, *n* (%)	180 (52.5)
Diabetes mellitus, *n* (%)	50 (14.6)
Coronary artery disease, *n* (%)	41 (12)

IQR, interquartile range; BMI, body mass index; FEV_1_, forced expiratory volume in 1 s; D_LCO_, diffusing capacity for carbon monoxide; ECOG, Eastern Cooperative Oncology Group.

**Table 2:** Operative details and outcomes.

Variable	All patients *N* = 343
Total robotic cases, *n* (%)	
Lobectomy	178 (51.9)
Segmentectomy	106 (30.9)
Wedge resection	59 (17.2)
Chylothorax, *n* (%)	3 (0.87)
Chest tubes removed by 12 h, *n* (%)	312 (91)
Home with chest tubes, *n* (%)	31 (9)
Estimated blood loss ml, median (IQR)	20 (10–20)
Operative time in minutes, median (IQR)	91 (75–110)
R0 resection, *n* (%)	343 (100)
Total lymph nodes removed, median (IQR)	29 (14–34)
Number of N1 stations, median	3
Number of N2 stations, median	5
30-day readmission, *n* (%)	3 (0.87)
30-day mortality, *n* (%)	0
90-day mortality, *n* (%)	0

IQR, interquartile range; ml, milliliters.

All patients were able to ingest the ice cream within 4 h of skin closure, with most consuming it in the recovery room. The median time to ingest was 1.5 h (range, 0.5–4 h). A total of three patients (0.87%) were diagnosed with a chylothorax postoperatively. Each patient was confirmed to have a chylothorax with a pleural fluid triglyceride level of >110 mg/ml. Two patients had noticeable white effluent drainage after consuming ice cream within 4 h of surgery. One patient had evidence of milky drainage identified on the morning of POD1, approximately 17 h after consuming ice cream. This patient's chest tube had only remained in place due to an ongoing air leak. One patient with a chylothorax had multistation metastatic N2 disease. The other two patients did not have positive nodal disease. None had induction therapy. Of the remaining 340 patients, none had a chylothorax diagnosed at any time postoperatively.

The three patients with chylothoraces went home on POD1 with their chest tubes in place. Their daily drainage of chyle was less than 450 ml. Given the low volume output, they were all managed medically with a no-fat, medium-chain triglyceride diet. Each chylothorax resolved within 6 days, and the chest tubes were removed in the office. The three patients with chylothoraces did not have any other minor or major complications within 90 days.

For the entire study cohort, chest tubes were removed within 4–12 h postoperatively in 91% of patients (312/343). Twenty-nine patients (8.5%) were discharged home with a chest tube on POD1, and two patients (0.58%) were discharged home with a chest tube on POD2. These 31 patients had their chest tubes removed on an outpatient basis within 2 weeks postoperatively. Five patients (1.5%) had postoperative procedures for pleural effusions (thoracentesis in four patients and pigtail catheter placement in one patient) within 1 month of surgery. Three patients were asymptomatic and had these procedures performed at their home institutions against our recommendations. None of these patients had chylothorax by report as the effluent was not cloudy and the triglyceride levels were low.

Minor morbidity occurred in four patients. This included atrial fibrillation in three patients and diffuse rash in one patient. There was no major morbidity and no 30- or 90-day mortality. Three patients were readmitted within 30 days for atrial fibrillation in two patients and shortness of breath in one patient. The patient readmitted with shortness of breath did not have a significant pleural effusion, and the workup for a pulmonary embolus was negative. There was no morbidity attributed to the diagnosis of a chylothorax.

Table 3 shows the efficacy of the recovery room ice cream challenge test. The total number of true positives was two, and that of false negatives was one. The ice cream test achieved a negative predictive value of 99.7% and a positive predictive value of 100%. The sensitivity, specificity, and accuracy of the test are 66.7%, 100%, and 99.7%, respectively.

**Table 3:** Efficacy of the recovery room ice cream challenge test.

	Chylothorax	No chylothorax	
Ice cream test +	2	0	Positive predictive value, 100% (2/2)
Ice cream test −	1	340	Negative predictive value, 99.7% (340/341)
	Sensitivity, 66.7% (2/3)	Specificity, 100% (340/340)	Accuracy, 99.7% (342/343)

## Discussion

Chylothorax is a rare but disconcerting complication after what was otherwise considered a well-performed minimally invasive operation by surgeons. Providing patients with ice cream in the recovery room is an inexpensive, safe, easily reproducible test to rule out chylothorax early after pulmonary resection with complete thoracic lymphadenectomy. The benefit of ruling out a chylothorax within a few hours after surgery is that the patients can have their surgical drain removed sooner, which we believe reduces pain and morbidity and improves patient experience. In this study, we found the recovery room ice cream challenge test has a high negative predictive value, positive predictive value, and specificity.

Robotic surgery, compared to video-assisted thoracoscopy, may lead to a more thorough lymph node removal and thus higher nodal upstaging due to improved visualization and accessibility of lymph node stations (13). Despite this, the incidence of chylothorax after robotic pulmonary resection with complete lymphadenectomy has remained low, as we have previously reported with a rate of 1.4% (1) and in this study with a rate of 0.87%. We favor and have systematically performed extensive and aggressive complete thoracic lymphadenectomy in all of our patients. We performed this even in those with suspected metastatic lesions or potentially benign pathologies because, unfortunately, frozen sections and preoperative clinical assumptions are often inaccurate. Furthermore, since we perform this procedure several times a day, we consider complete thoracic lymphadenectomy to be very safe, even on the left side near the recurrent laryngeal nerve.

We have significantly lowered our reported incidence of chylothorax over time through improved operative techniques and hypervigilance with several specific moves. For instance, we perform the complete thoracic lymphadenectomy first, which allows for chylous drainage to be evident when we come back to look at the nodal beds after completion of the pulmonary resection. In addition, we carefully look and often clip any obvious clear channels that we see in the base of the 2R lymph node dissection. This of course is more readily seen with the robotic platform which utilizes 10 times 3D magnification and often identifies structures that may have been missed during video-assisted thoracoscopic or open surgery.

Chylothoraces that occur are commonly a result of injury to collaterals during dissection of N2 mediastinal lymph nodes, rather than injury to the main thoracic duct. For this reason, the majority of patients with chylothorax after pulmonary resection, as compared to esophagectomy, for example, will observe resolution of the chylothorax within days by simply modifying their diet (1). If they have metastatic nodal disease, particularly N2 disease, it is less likely to resolve with diet alone, probably because of lymphatic congestion, and oftentimes systemic therapy may be needed (1). This aligns with our finding as reported here, since all three patients with chylothorax improved within 6 days on a modified diet.

We observed one false-negative result in a patient whose chylothorax was diagnosed more than 12 h after consuming ice cream in the recovery room. We are unsure why the test was falsely negative since the patient consumed all of the prescribed ice cream within the allotted time. The chest tube in this patient remained in place due to an ongoing air leak; otherwise, we would have removed it. Given our standard of practice to remove chest tubes within 4–12 h postoperatively (now we are prospectively studying the removal of chest tubes prior to leaving the operating room in the majority of our lobectomy and segmentectomy patients), it is possible that we may have missed chylothoraces in other patients whose chest tubes were removed early. If so, these chylothoraces in these patients seemed to be inconsequential or clinically insignificant, as no patients in this series returned with a chylous pleural effusion after being discharged home. Although the test failed to detect one chylothorax, perhaps it may still effectively detect the clinically significant chylothoraces. While we do not know the answer to this, we can confirm that no patient in this series developed a symptomatic chylothorax that required drainage. Additionally, we provided patients with a 106 ml (3.6 fl oz) cup of ice cream because that is what was available at our institution. The optimal quantity and type of ice cream and time and venue (we chose the recovery room to speed removal) to deliver the ice cream have not been studied by us and thus remain unknown.

There are several limitations to this study. First, it reflects the experience of only a single surgeon, and we have already had many process improvements intraoperatively to reduce our reported incidence of chylothorax. Our reported low incidence of chylothorax may affect the efficacy of the ice cream test. Second, the false-negative rate may be inaccurate or underreported as some patients may have had chylothorax that was missed but clinically insignificant, since in those patients to whom we direct their 90-day postoperative care, we do not perform thoracentesis for postoperative effusions unless they are truly symptomatic. The vast majority of these are monitored rather than treated.

A strength of this study is that it consists of a consecutive series of patients without preselection. All patients were offered a robotic platform and underwent robotic lobectomy, segmentectomy, or wedge resection with complete thoracic lymphadenectomy and complied with eating the ice cream. Additionally, the intervention in this study was simple and inexpensive without the burden of significant resource utilization and thus easily scaled and reproduced. In fact, all thoracic surgeons at our institution have now adopted this practice and have achieved success with it. It is now our standard of practice in patients for whom we leave chest tubes postoperatively.

## Conclusion

In conclusion, providing patients with ice cream in the recovery room after robotic pulmonary resection with complete thoracic lymphadenectomy is a safe and inexpensive method to reliably rule out chylothorax within a few hours after surgery.

## Data Availability

The raw data supporting the conclusions of this article will be made available by the authors, without undue reservation.
